# ‘For a mere cough, men must just chew *Conjex*, gain strength, and continue working’: the provider construction and tuberculosis care-seeking implications in Blantyre, Malawi

**DOI:** 10.3402/gha.v8.26292

**Published:** 2015-03-31

**Authors:** Jeremiah Chikovore, Graham Hart, Moses Kumwenda, Geoffrey A. Chipungu, Liz Corbett

**Affiliations:** 1 HIV/AIDS, Sexually Transmitted Infections & TB, Human Sciences Research Council, Durban, South Africa; 2 School of Life & Medical Sciences, University College London, London, United Kingdom; 3 Helse Nord TB Initiative, College of Medicine, Blantyre, Malawi; 4 Malawi Liverpool Wellcome Research Programme, Blantyre, Malawi; 5 London School of Hygiene and Tropical Medicine, London, United Kingdom

**Keywords:** Malawi, masculinity, tuberculosis, healthcare seeking, gender, provider, qualitative, low income

## Abstract

**Background:**

Delay by men in seeking healthcare results in their higher mortality while on HIV or tuberculosis (TB) treatment and contributes to ongoing community-level disease transmission before going on treatment.

**Objective:**

To understand masculinity's role in delay in healthcare seeking for men, with a focus on TB-suggestive symptoms.

**Design:**

Data were collected between March 2011 and March 2012 in low-income suburbs in urban Blantyre using focus group discussions with community members (*n*=8) and health workers (*n*=2), in-depth interviews with 20 TB patients (female=14) and 20 uninvestigated chronic coughers (female=8), and a 3-day participatory workshop with 27 health stakeholder representatives. The research process drew to a large extent on grounded theory principles in the manner of Strauss and Corbin (1998) and also Charmaz (1995).

**Results:**

Role descriptions by both men and women in the study universally assigned men as primary material providers for their immediate family, that is, the ones earning and bringing livelihood and additional material needs. In a context where collectivism was valued, men were also expected to lead the provision of support to wider kin. Successful role enactment was considered key to achieving recognition as an adequate man; at the same time, job scarcity and insecurity, and low earnings gravely impeded men. Pressures to generate continuing income then meant constantly looking for jobs, or working continuously to retain insecure jobs or to raise money through self-employment. All this led men to relegate their health considerations.

**Conclusions:**

Early engagement with formal healthcare is critical to dealing with TB and HIV. However, role constructions as portrayed for men in this study, along with the opportunity costs of acknowledging illness seem, in conditions of vulnerability, important barriers to care-seeking. There is a need to address hidden care-seeking costs and to consider more complex interventions, including reducing precarity, in efforts to improve men's engagement with their health.

Tuberculosis (TB) is a leading cause of adult morbidity and mortality globally. In 2012, it was responsible for 8.6 million cases and 1.3 million deaths of which 0.32 million deaths were in people living with HIV (1). Although TB rates have been declining globally, the World Health Organization (WHO) considers the rate of decline to be slow and the 50% target for reduction of active cases in the community by 2015 unlikely to be met (1). In particular, the European and African regions are unlikely to meet prevalence and mortality targets (1). In addition, the African continent is over-represented among TB cases: with only 14% of the world population, it accounted for 27% of global TB cases in 2012, coming second only to Asia (1), and effectively having the largest per capita TB rate. The resurgence of TB in Africa has been attributed to a combination of weak health systems, rapid urbanisation, poor living conditions in fast-growing cities, and the HIV epidemic (2).

Engagement with formal healthcare has become increasingly crucial, particularly with respect to TB and HIV/AIDS. HIV treatment is now known to have a preventive effect (3–9) : of an estimated 90% of people in sub-Saharan Africa who know their HIV status and are on treatment, 76% have achieved suppressed viral load and are thus unlikely to transmit HIV to a partner (4). Similarly for TB, a person going into treatment ceases being infectious after 2 weeks, whereas one who stays undiagnosed and untreated infects an estimated 10–15 others in the community per year (10).

The majority of people in sub-Saharan Africa who need treatment for HIV are, however, not accessing it (4). In addition, 3 million cases of TB went undiagnosed in 2012 (1). Being male is specifically a risk factor for late HIV and TB diagnosis and treatment, as well as death while on treatment (1, 4, 8, 9, 11–13) . Despite men's key role in TB transmission dynamics, relatively limited emphasis has been put on their epidemiological or social positions. When a gender perspective is incorporated into policy or research, the focus is often on women (e.g. 1, 14–18) . Men are evidently less well served by health services, given substantial investments therein over the past decade. Without a more effective male inclusive approach, men will continue to serve as a major reservoir of ongoing TB transmission at community level. Given, moreover, the ways rapid and drastic socio-economic and structural changes are reshaping gender and social relations (19, 20), the present study sought to understand masculinity's role in TB-related healthcare seeking in a contemporary low-income urban setting. The long-term goal was to develop candidate interventions targeting men in TB control.

The paper is guided by a framework that draws from three related approaches within the gender and social science literature. One approach, the *social constructionist* perspective, holds that women and men think and act in the ways that they do, not because of their psychological traits but because of concepts about femininity and masculinity that they adopt from their culture; hence, gender is, from this perspective, ‘a dynamic social structure’ (21, p. 1387). The second approach, *gender relational theory*, as described by Connell, sees gender as a multidimensional structure operating in a complex network of institutions (22). In Connell's view, gender thus entails what women and men do towards each other and against what the other sex does, and as played out on world scale, interwoven with the history of colonialism and contemporary structural effects of globalisation (23). Connell stresses the multiple, hierarchical, and contradictory nature of masculinity (24). In southern Africa, socio-economic changes have shaped masculine behaviour and sexual practices on mines, farms, and in cities, giving rise to the male provider role as a defining feature of manhood and fatherhood, and driving masculine behaviours such as violence on self and others, and excessive alcohol consumption (20, 25–30) . The third approach, *hybridity*, describes the intersection of macro-structural forces with cultural and local factors, generating ways of being and relating that are defined by flux, identity searches, mixing of cultures and signals, contradictions, and split consciousness (31). Connell states that even though, as derived from postcolonial theory, hybridity signifies diversity, there is need to stress ‘the devastating colonial histories of forced disruption’ (22, p. 65). The paper also borrows from the widely described concept of ‘masculinity crisis’, applying it here in the sense of the struggles men seem to go through existing within wide-ranging and drastic changes that are dismantling familiar roles.

## Methods

### Setting

The study was carried out in Malawi, a low-income agriculture-dependent country with a population of 15 million, two-thirds of whom lived below the poverty datum line in 2010 with a further 23.4% vulnerable to or at risk of becoming multi-dimensionally poor (32). Ninety per cent of the people in Malawi are considered to have some link to informal employment (33). The country has one of the fastest rates of urbanisation globally (34), and 70% of the population of Blantyre, the study city, live in unplanned settlements (35). The study was carried out in three high-density locales within these settlements. Adult national HIV prevalence is estimated at 10.8%; antiretroviral treatment coverage at 69% based on 2010 guidelines (36); TB incidence at 163/100,000, of whom 78% are diagnosed within a year against the global target of 70% (36); TB case notification rate for Blantyre city at 458/100,000 of whom 60% were men and 40% women (Dr EL Corbett, personal communication), and treatment success rate at 85% (1).

### Design, participants, and sampling

Given limited understandings and the complexity around the research topic, we triangulated methods and data sources, and purposefully chose the sample and varied it by sex to explore different dimensions while including appropriate participants (37, 38). Chronic cough was used as the main entry point to the study [for detailed explanation of the justification, see (39)]. Data were collected between March 2011 and March 2012, using mixed- and single-sex focus group discussions (FGDs) with 74 ordinary community members and 20 health workers (HWs), in-depth interviews (IDIs) with 20 TB patients (female=14) and 20 uninvestigated chronic coughers (female=8), and a 3-day participatory workshop with 27 health stakeholder representatives. The research process was aligned to grounded theory as propounded by Strauss and Corbin (40) and Charmaz (41). According to Charmaz

… you start with individual cases, incidents or experiences and develop progressively more abstract conceptual categories to synthesize, to explain and to understand your data and to identify patterned relationships within it. You begin with an area to study. Then, you build your theoretical analysis on what you discover is relevant in the actual worlds that you study within this area. (41), p. 28)

**Fig. 1:**
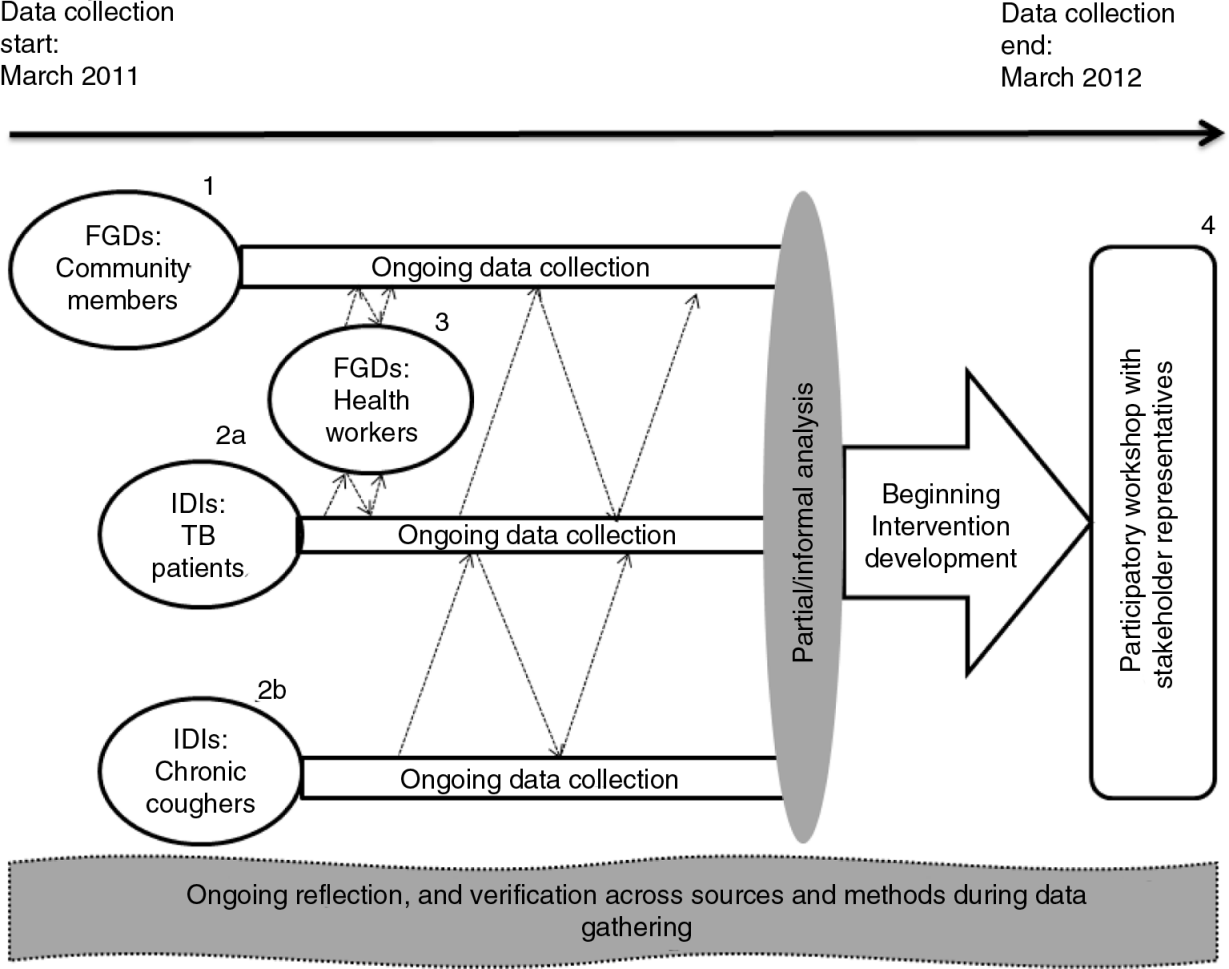
The data collection process and data sources.

The study questions and insights about gender roles and relations that we brought to the study informed our decisions regarding the initial samples as well as the content of the tools. More specific questions emerged during fieldwork and tools were modified to follow emerging crucial leads. The research process was in this sense reflexive and nonlinear (40, 41). Community member FGDs were initiated first, followed shortly by IDI commencement, and then by HW FGDs as a once-off activity. The workshop was done last and intended to begin the process of developing potential interventions (Fig. 1 and Table 1).

**Table 1:** Participants and techniques and their justification, and respective sample characteristics

Participants (technique, sample characteristics)	Primary exploratory outcomes	Justification of choice of method and participants
Uninvestigated chronic coughers (IDIs; *n*=20; *F*=8; *M*=12; age range 18–77; average age=36; balanced by marital status)	Experiences of symptoms; steps taken and reasons; reasons for not being investigated	The stigma of TB/cough/HIV, and personal data require private interview setting; IDIs target information about individual experience.
Newly diagnosed TB patients (IDIs; *n*=20; *F*=14; *M*=6; age range 21–70, average age=33; slight majority married)	Experiences of symptoms; steps taken and reasons; care-seeking experiences	The stigma of TB/cough/HIV, and personal data require private interview setting; IDIs target information about individual experience.
Community members (FGDs; *n*=8; Male only=3; Female only=3; Mixed sex=2; Total no. of participants=74)	General beliefs about gender, health, and care-seeking behaviour; perceptions of cough and TB symptoms, and health services	FGDs help generate information on beliefs and norms; They aid exploration as participants debate and contradict each other (42, 43)
Health workers (FGDs; *n*=2; both mixed sex; no. of participants=20)	Views on 1) men's care-seeking, 2) interface of health services with community	As for community FGD above; Also, participants were expert informants based on their special knowledge.
Health stakeholders (participatory workshop; 27 participants; *F*=14; *M*=13)	Develop candidate interventions; discuss acceptability of proposed interventions	Participants were experts in different domains related to health [see (39)]

FGDs=focus group discussions; IDI=in-depth interview; F=female; M=male.

Two local social science graduates including (MK), both fluent in Chichewa, collected the FGD and IDI data. We set minimum sample sizes for the different categories of participants *a priori*, taking into consideration the numbers deemed adequate to explore phenomena in qualitative research (44). Informal analysis during and after the first round of data collection led to a determination that a fairly complete picture had been generated, and no significant additional information would be generated by further data collection. [The methods, including approaches to recruitment, are also described elsewhere, see (39).]

### Ethics

Malawi College of Medicine and Human Sciences Research Council research ethics committees approved the study. We sought and received clearance to enter the communities from executive leaders of Blantyre and local leaders. The district medical officer and facility managers also granted permission to access patients’ records. Written informed consent and permission to record data were sought from participants, and anonymity and confidentiality were maintained at all stages of the research. Participants were provided refreshments and reimbursed their transport costs.

### Analysis

Focus group and IDI data were recorded, transcribed, and translated by trained personnel; checked for accuracy by (MK); and for intelligibility by (JC), who is fluent in a related regional language, Shona. The transcripts were entered into NVivo data analysis software (Version 8, QSR International Pty. Ltd., 2008) and open coded partly using predetermined questions but largely inductively through identifying emerging concepts. In the process of coding and abstracting from the data, concepts were classified according to their properties, and the emerging categories reviewed and refined through the coding process and as they were related to each other and to emerging theory (40, 41).

## Results

Emerging themes presented separately included the manifestation of masculinity as control and the healthcare seeking implications, masculinity-mediated links made between TB and HIV, and health system barriers affecting women and men generally. This paper focuses on another theme elucidated during the study: men's material provider representation. Role descriptions by both men and women in the study universally assigned men as primary material providers for their immediate family, that is, the ones earning and bringing livelihood and additional material needs. In a context where collectivism was valued, men were also expected to lead the provision of support to wider kin. Successful role enactment was considered key to achieving recognition as an adequate man; at the same time, job scarcity and insecurity, and low earnings gravely impeded men. Pressures to generate continuing income then meant constantly looking for jobs, or working continuously to retain insecure jobs or to raise money through self-employment. All this led men to relegate their health considerations.

### A material provider representation for men in conditions of economic difficulty

#### Role delineations and accompanying tensions in men and families

Women and men affirmed the gender role distinction that assigns men the task of earning cash income and women responsibility for managing the domestic domain. Men were held responsible for their immediate families’ entire breadth of material requirements. Participants in a community women's FGD described the responsibility as involving ‘ensuring that a woman gets all her needs’, ‘finding food for us’, ‘buying us clothes’, ‘building a house for us’, ‘upon children entering high school … sends fees fast … and uniform too’.

The role's emergence and basis within the political and economic context of colonial Southern Africa is well documented [see also (39)]. The role, however, persists even when men's earning opportunities have significantly diminished, and women increasingly participate in income generation (20, 30, 45–47) . Women in this study described, for instance, the importance of ‘helping’ husbands with income generation, and many mentioned ‘doing businesses’. Their income was nevertheless considered supplementary, and men the ones chiefly and ultimately responsible for households’ domestic and external financial needs and obligations.

… all relatives expect a man to see that his family has a good house, good toilet, storeroom full of food; the children are in school, the wife is well taken care of, and relatives needing assistance are getting it. (Community men's FGD)

The accounts then illuminated stress and tensions around acquiring, managing, and sharing resources where they were simply scarce. The collectivism that characterises African social systems (48) meant, for instance, that being present around and benevolent towards kin was valued highly. Furthermore, helping out was treated as obligatory in the case of relatives such as in-laws, while the unstable and precarious economy also impelled cooperation to guard against future eventualities. In view of the pressures (‘It is my responsibility … I have to do it. If I have problems, I borrow’ – male TB patient), immense stress was experienced due to scarcity of resources coupled with inordinate demands from kin.

Relatives, you don't do what they expect, they call you bad … both wife and husband you are ‘bad’…. You also may not receive help when a problem comes your way. (Community men's FGD)

My relatives expect a lot…. You give that little when people bring up a small problem. But with this cough, I don't get money every day … when you give, people assume you have more … not knowing you're just trying … It depresses me a lot. (IDI, 21-year-old informal clothes trader, male TB patient)

In particular, being responsible for large numbers of dependants while living under harsh economic conditions and beset with poor health (as the participant above also illustrates) made men's expectation to provide materially burdensome.

… things aren't going as I wish. I get money from my *business* but not enough (to) help at the right time. … What I get … is little, but my responsibility huge. (IDI, married 41-year-old chronic cougher, father of four; self-employed carpenter)

… My wife doesn't work … my elderly mother stays with us … I have a young brother in high school … all five of us together expecting food … clothes … Problems at the village too are my responsibility. … Any minute something pitches … maybe at my in-laws’ … and my wife just glares at me. (IDI, married 30-year-old mother of two and TB patient)

The urban milieu and its attendant flux and ‘competitive materialism’ (49), p. 41), furthermore, regularly shifted needs and desires of families, with men then expected to bring their families at par with others’ attainments or risk becoming subject of wrath and contempt.

We get to be envious: ‘Oh my, this one, her house! Mine is nowhere near that … And that one, God blessed her with a *screen* (television), she has everything, but I don't.’ (Woman in mixed-sex community FGD)

We look at how our friends in other families live, maybe they dress and eat well, and we ask, ‘Why does this husband of mine behave like this? Nothing at all like dressing well in our family’ … so you become *weak* (demoralised), just from looking at and admiring that friend of yours. (Community women's FGD)

#### Ambivalence and contradiction in general life experiences, and in perspectives of men's failure

The accounts portrayed general conflict, struggle, and contradictions, as people seemed torn among multiple worlds. The value put on assisting kin materially in a treacherous economy, for instance, coexisted with growing pressures emanating within the same economy to adopt more individualistic lifestyles. Similar ambivalence was expressed when women described their sympathy for men's situation arising from being saddled with impossible demands while already overwhelmed.

We make this man carry too much responsibility … saying, ‘Everything will be done by him’ … But it's often difficult for men. In the end, it appears there's no love in the family…. (Community women's FGD)

… in terms of money … it gets *tight* (difficult) for him. … He then looks for a chance to run … from his responsibility, leaving the woman and children … why? Because we're making him carry all the responsibility. (Community women's FGD)

The women then turned around and castigated men for lacking initiative, being irresponsible with money, and being addicted to liquor. In women's view, notwithstanding the adverse circumstances, men abetted and were largely responsible for their own failure.

It's not all about being employed … Most get money, spend on *mowa* (liquor) and then sleep, come morning, wake up and just sit. Meanwhile their peers are doing *business*, supporting their households and children…. (30-year-old married businesswoman in a mixed-sex FGD)

… once they lay hands on a MK100 (usd0.30) note, they're immediately on *Rider*. *Ten Rider*, they're sorted. To then think ‘How do I support my family?’ they can't. … just always drunk. (24-year-old unmarried businesswoman in women only FGD)

Women exhorted men to look harder for jobs, take up any that arose, and work without respite. Their tone and emphasis seemed to give men little room to consider health ahead of earning.

He just must have money … work here and there, doing something, any piecework … sweeping … digging latrines … whatever is available …. Someone asks ‘Please help thatch my house’, and that way he gets money …. Going up the mountain to fetch firewood to sell; because he must always think: What will my children eat? Buying and selling bananas ‘Just so my children get something to eat.’ … Even touting at taxi ranks. (Community women's FGDs)

The accounts also described the treatment and experiences of men who failed. Failure seemed especially grave when visible to the public world, for example, when one's children cried persistently and pestered neighbours, ‘showing that as a man you are failing your responsibility’ (Community men's FGD). The men were said to be humiliated, devalued socially and shunned by peers, and abused psychologically in and out of home.

They're in big trouble, don't receive respect and seen as useless … you lead an isolated life without friends, your marriage breaks … and your own children won't respect you. (Man in mixed-sex community FGD)

Those men are humiliated, starting in their families all the way to outsiders and relatives … to the point of being denied food in their homes and teased when walking the street. (Woman in mixed-sex community FGD)

### ‘Because of this need and heart to work hard, a man appears to be strong because he keeps working even while sick’

More direct connections were drawn between men's role construction as material provider and their health response. It was stressed, for instance, that as heads of and also sources of inspiration and income to their families, men must not promptly acknowledge illnesses particularly ‘minor’ ones. Minor illness was illustrated with ‘headache’, *chimfine* (‘flu’ said to be treatable by drinking a lot of water), ‘stomach ache’, ‘mere cough’ (described as any cough ranging from under 3 days’ to 3 weeks’ duration, or responding to self-medication), *kungomva kuzizira* (‘feeling a bit cold’ ‘fever’), and *kathupi kakutentha pang'ono* (‘mild aches’). Overlooking what clearly seemed to be vague and largely speculative descriptions of what constitutes minor illness, men were expected by both men and women to either ignore such illness or self-medicate, and continue functioning and fending for their families.

… the whole family looks up to him … It can't be whenever he has a headache, he jumps in bed. … Things in his house would stall. (Community women's FGD)

“… We look up to the man to bring. If he's weak against minor diseases, everything else stops. If it's a headache, they must take *Aspirin*; for this mere cough they simply chew *Conjex*, gain strength and continue with the work they must do. (Woman in community mixed-sex FGD)

Interestingly, contrary to views that men lacked the motivation to work, it seemed many already pursued earning even to the extent of overlooking their health. In addition, the images of power and agency often associated with men were absent, and the men in this study appeared to be shunted by an unmanageable drive to earn money and retain insecure jobs, with no control over their lives.

He'll say he's sick yet still go around, looking for things. He even tells his wife his body is not well, but still starts off for work, because of thinking about his responsibility in the home. The woman remains behind, anxious … only to see him return in the evening; still saying he's not well. (IDI, 46-year-old widow and TB patient)

A chronic cougher described how he was dragging himself every day and working long hours, sparing no time to be investigated although he seemed ready.

I just haven't had time … been very busy working, so I just push myself on – coughing. I leave in the morning around *past five*, and return only around *seven* … including Sunday and Saturday …. So really, I'm just thinking they should … do the process and find out if it is TB. (IDI, 37-year-old, 2-month chronic cougher)

Men's precarious employment situation and difficulties balancing failing health with keeping insecure jobs were further demonstrated when a former metered-taxi driver recounted the events leading to his dismissal, which were triggered by missing a day of work due to illness. His description that his health plummeted shortly afterwards suggests the possible effects of unemployment-related stress but also that he had perhaps nursed the illness for some time.

He (my boss) was wondering where I still was … then the other drivers around told him I'd drunk so much the previous day I wouldn't make it to work. Furious, he told me never to touch his car ever again … But two weeks later, everyone could see I was *finished* (wasting). (IDI, married male 30-year-old father of two, TB patient)

Under immense pressure to earn while unwell, men resorted to painkillers just to manage but also avoid taking ‘expensive’ breaks from work. The distantly located primary care facilities made it even less convenient to initiate formal care-seeking.

If I focus on this sickness's pain, then I won't work and achieve what I want. I'd rather get *Panado* to relieve the body pains and continue with my work. (IDI, 41-year-old married father of four, self-employed carpenter, chronic cougher)

… Men don't like going to the hospital; they see it like wasting time instead of working. (IDI, 38-year-old father of one, carpenter, chronic cougher)

## Conclusions

The connection between men's material provider concept and their healthcare seeking behaviours is rarely addressed in the literature from the African continent, a scenario attributable in part to the prior focus within the gender and health field on women. In this study of men's care-seeking delay, we had therefore not expected the role to emerge in the way it did; rather, we had expected that masculinity would manifest through commonly documented representations such as power, agency, strength, and invulnerability. Although the signifiers emerged in the present study, they assumed more complex forms than had been foreseen.

In some settings in Africa, the provider role for men has been linked to colonial intervention and especially the measures instituted to extract cheap labour for mines and farms (26, 30, 50, 51) which consolidated the cash economy while simultaneously restricting access to the cash to men. In the process, ‘the rural, domestic, feminine, and non-monetised became devalued and synonymous with poverty’ (39). Earning and paid work continue to be symbols of status and prestige – hence competent masculinity – in present day, partly due to powerful forces related to globalisation. Access to them is nevertheless considerably limited for most men. Many countries on the continent including Malawi, moreover, make little or no provision for paternal leave (52, 53), essentially endorsing the separation of men from domestic spaces and tasks. Although Malawi's employment policy opposes discrimination on the basis of parenting status, it has been reported that men do not use the provision to become more involved domestically as this contravenes dominant masculinity stereotypes (53). With some tacit backing from policy, therefore, men continue to be tied to a material provider construction even when it is increasingly difficult for them to fulfil it.

Threats to achieving desired masculinity representations are reported to fuel in men intensified efforts to prove that they (can) meet the grade; alternatively, men redirect their focus to more accessible versions which they still proceed to enact to extremes (20, 21, 28, 54). In other words, the more men are involuntarily swayed towards a greater presence and participation in the domestic sphere (and, therefore, further away from the public sphere encompassing work and earning), the greater their effort to dissociate from it. The ‘flight from the feminine’ (55), p. 122) may take the form of seeking to succeed in work and in the material provider image (55, 56) at all cost even if this means sidelining their own health.

In this study, the urban setting and associated consumerism are portrayed exerting pressure on women, who in turn relay it to men helping shape the criteria by which the latter's adequacy is assessed. Furthermore, when struggling to meet basic survival requirements, families and men are forced to make pragmatic choices, in this case dissuading able-bodied men in the households from seeking healthcare or even acknowledging illness until it is unequivocally determined to be serious. There seems to be a paradox thus, whereby the same power and control men are documented to wield over resources, and which they reportedly use to dictate women's and children's access to care, also affects men's own utilisation of care. Ultimately, though, the construction of men's health behaviours within gender relational dynamics (23) means that efforts to engage men in health must not exclude women.

Literature frequently mentions men's provider role in the context of immediate families. African social systems, however, stress communality and extended family form (48), although the values are shifting within socio-economic changes. The same changes, nevertheless, also engender collectivism as a form of social and economic security (57), meaning that families (and men) are compelled to share scarce resources. The details surrounding resource flows across households fall beyond the present study's scope. What is clear, though, is that for men and families in low-resource communities, assisting kin is at once desirable and experienced as burdensome. Households that are drawn (deeper) into indigence owing to shedding of resources to kin, furthermore, risk facing more diminished choices around their members’ engagement with health.

Men, families, and communities seem to exist in conditions of ‘hybridity’, under a milieu characterised by tension, struggle, and ambivalence, amid widespread economic hardship all largely linked to globalisation and related socio-economic changes. It is possible this scenario partly explains the alcohol abuse described for men in this study, which may be important given studies report men using alcohol to suppress illness pain and symptoms (58).

The present findings also need to be located in the context where international efforts to deal with HIV and AIDS – and by extension TB – are placing greater emphasis on early engagement with formal care, and on groups that are over-represented among those left out of care (4). Frequently, men are considered to avoid healthcare because it contravenes sought-after masculine representations (21, 59, 60). In illuminating relational and structural factors that affect men's health-related responses, this study points to limitations in approaches that indiscriminately blame men for their behaviour, or explain it in general terms such as ‘stoicism’. The study suggests a need for interventions targeting men to assume complex outlooks, including reducing social precarity for men, families, and communities. A limitation of the study is that it is based on a non-probability sample, and is therefore not statistically generalisable in the context of Malawi or the region. It is nevertheless possible, in this type of inquiry, to transfer findings to contexts considered similar to the study setting.
